# Association Between Animal-to-Plant Protein Intake and Low Skeletal Muscle Mass Among Lebanese Older Adults

**DOI:** 10.3390/nu18203333

**Published:** 2026-10-12

**Authors:** Cynthia El Hajj, Stéphane Walrand, Mariana Helou, Souheil Hallit

**Affiliations:** 1 Department of Nutrition and Food Science, School of Arts and Sciences, Lebanese American University, Beirut 1102, Lebanon; 2 Nutrition and Dietetics Department, Saint Charles Hospital, Beirut P.O. Box 40–50, Lebanon; 3 Unité de Nutrition Humaine (UNH), INRAE, CHU Clermont-Ferrand, Université Clermont Auvergne, F-63000 Clermont–Ferrand, France; 4 Department of Medicine, School of Medicine, Lebanese American University, Beirut 1102 2801, Lebanon; 5 School of Medicine and Medical Sciences, Holy Spirit University of Kaslik, Jounieh P.O. Box 446, Lebanon; 6 Applied Science Research Center, Applied Science Private University, Amman 11937, Jordan

**Keywords:** skeletal muscle mass, older adults, animal protein, plant protein, handgrip strength, physical performance, Lebanon

## Abstract

Background/Objectives: Sarcopenia is a major age-related health condition characterized by loss of muscle mass, strength and physical performance. Adequate protein intake is essential for maintaining muscle health, but the role of protein sources and particularly animal-to-plant protein ratio remains unclear. This study aimed to investigate the association between dietary protein sources, particularly animal–plant protein ratio, and low skeletal muscle mass among Lebanese older adults. Methods: A cross-sectional study was performed on 162 older adults aged >60 years using a food frequency questionnaire (FFQ) to assess dietary protein intake. Protein sources were classified as animal- or plant-based. Skeletal muscle mass index was calculated as skeletal muscle mass/height^2^, and cut-off thresholds for sarcopenia were set at 7.26 kg/m^2^ for men and 5.45 kg/m^2^ for women. Handgrip strength and the Short Physical Performance Battery (SPPB) were used to assess muscle strength and physical performance. Associations between protein intake, protein source, and low skeletal muscle mass were evaluated using appropriate statistical analyses. Results: Frequency of low skeletal muscle mass was 49.4%. Logistic regression analysis showed that higher handgrip strength (aOR = 0.92), higher SPPB (aOR = 0.49) and higher intake of animal protein (aOR = 0.87) and plant protein (aOR = 0.88) were significantly associated with lower odds of low skeletal muscle mass. Conclusions: Higher intakes of both animal- and plant-source protein were associated with lower odds of low skeletal muscle mass among Lebanese older adults. Animal-source protein showed a somewhat stronger association than plant-source protein in the present analysis. However, because of the cross-sectional design, these findings should be interpreted as associations and do not establish causality. Longitudinal and intervention studies are needed to clarify whether dietary protein source contributes to changes in skeletal muscle mass and muscle function in older adults.

## 1. Introduction

Sarcopenia is characterized by involuntary loss of skeletal muscle mass and muscle strength with aging. It may start as early as the fourth decade of life, and the related decline can reach up to 50% by the eighth decade of life [1]. Sarcopenia has numerous consequences that are often associated with increased risk of fractures and frailty, which can in turn contribute to functional impairment and disability and other adverse health outcomes [2,3,4]. Sarcopenia ultimately increases the risk of mortality among older people. Numerous factors have been linked to the development of sarcopenia, including unfavorable genetics and heredity, lack of physical activity, low dietary intake of protein [5], and subclinical inflammation inducing insulin and anabolic resistance [6].

One strategy to address the age-related decline in skeletal muscle mass in older adults is to increase protein intake to preserve protein balance [7], i.e., the balance between protein synthesis rate and protein degradation rate, to maintain muscle mass and consequently slow the progression of sarcopenia [7]. Dietary animal protein is known to contain higher levels of essential amino acids than dietary plant protein, and could thus better sustain muscle protein synthesis [8]. Furthermore, animal-source proteins contain all the essential amino acids whereas plant-source proteins are deficient in one or more essential amino acids. For example, cereals are low in lysine relative to human nutritional requirements, and legumes are deficient in sulfur-containing amino acids. Note, however, that these deficiencies in essential amino acids in plant proteins can be overcome by using a blend of plant proteins that together compensate for the individual protein deficits in essential amino acids.

However, there is no clear evidence that protein or amino acid supplementation increases muscle mass and muscle strength in healthy older adults [9,10], and research investigating the association between protein intake and muscle mass in older adults has yielded inconsistent results. Some studies have found that consuming animal and mixed plant proteins increases muscle mass [11], whereas other studies found no effect of protein source, particularly when these proteins were combined with resistance training [12,13].

Animal protein was shown to be more effective at preserving skeletal muscle mass than plant proteins [14,15]. Previous studies have shown that adults over 50 years of age with a high intake of animal proteins have a higher muscle mass, lower risk of functional impairment, and greater maintenance of grip strength [14,16]. Daly et al. showed that a diet enriched with lean red meat is capable of considerably improving total body and leg lean tissue mass and leg muscle strength in older women [17]. Korat et al. reported that higher plant protein intake was associated with higher odds of healthy aging [18]. A recent systematic review and dose–response meta-analysis of prospective cohort studies found that higher total protein intake and higher plant-based protein intake were associated with a lower risk of frailty among older adults [19]. However, a recent study showed no differences in muscle mass and strength between a lacto-ovo-vegetarian diet and a beef-containing diet in older men following a 12-week strength training program [15].

Here, to resolve this issue, we examine the association between dietary protein, particularly the link between animal protein and plant protein, and muscle mass among the Lebanese older-adult population. Lebanese older adults can obtain a relatively large proportion of their protein from plant-based sources, including legumes and cereals, compared with other populations in which a larger proportion of dietary protein is animal-source. Although plant proteins contribute to a considerable share of overall protein intake, differences in essential amino acid composition, leucine content and digestibility may influence their capacity to stimulate muscle protein synthesis. Thus, the aim of this study was to evaluate the relationship between dietary protein intake, in particular animal–plant protein ratio, and skeletal muscle mass, and thus the development of sarcopenia in Lebanese older adults.

## 2. Methods

### 2.1. Design and Participants

This study was a cross-sectional study on low and normal skeletal muscle mass among Lebanese older adults. A total of 162 older adults aged >60 years were recruited from Saint Charles Hospital in Beirut and its affiliated geriatric care center between June and October 2025, using a convenience sampling approach. Eligible older adults attending or referred to the hospital or geriatric care center or volunteering to participate were invited to take part in the study. Recruitment was conducted irrespective of low muscle mass status, and no predefined proportion or quota of participants with or without low muscle mass was established. Low muscle mass status was assessed after recruitment according to the study criteria. Skeletal muscle mass was estimated by bioelectrical impedance analysis (BIA) (Tanita MC−780 MA). Skeletal muscle mass index was calculated as skeletal muscle mass/height^2^, and the cut-off thresholds set for sarcopenia were 7.26 kg/m^2^ for men and 5.45 kg/m^2^ for women [20].

It is important to clarify that these criteria were used to classify low skeletal muscle mass, rather than to establish a complete diagnosis of sarcopenia according to contemporary multidimensional diagnostic algorithms. Handgrip strength (HGS) and physical performance, assessed using the Short Physical Performance Battery (SPPB), were measured as complementary indicators of muscle strength and physical performance but were not used as independent criteria for the primary classification of low skeletal muscle mass. Thus, throughout this manuscript, the primary outcome is referred to as low skeletal muscle mass rather than sarcopenia unless referring specifically to the broader clinical condition or to findings from previous studies.

Subjects were included if they were aged >60 years old and sarcopenic (for the sarcopenic group) and could complete the required tests, anthropometric measurements, and food frequency questionnaire (FFQ). Potential participants were excluded if they presented severe cognitive impairment and/or severe heart, liver and/or kidney dysfunction [14]. The FFQ was specifically developed for this study and included 80 food items reflecting foods commonly consumed by the Lebanese population, with a particular emphasis on major sources of animal and plant protein. Participants were asked to report their usual dietary intake over the course of the previous 3 months. The FFQ was translated into Arabic for administration to the Lebanese study population. During development of the FFQ, the food items were reviewed by nutrition experts and adapted to reflect commonly consumed Lebanese foods. The Arabic version was pilot-tested among Lebanese participants to assess clarity and comprehension. In addition, FFQ-derived dietary intake was compared against dietary intake data obtained from 24 h diet recalls to assess the relative validity of the FFQ. Physical activity was assessed through self-reported information on participants’ usual level of regular exercise. Participants were categorized as physically active or inactive based on regular engagement in moderate-to-vigorous physical activity versus no regular exercise. Informed consent was obtained from all subjects involved in the study. All study procedures were conducted per the Declaration of Helsinki ethical principles for medical research involving human subjects. All protocols were approved by the institutional review board for Saint Charles Hospital in Lebanon.

### 2.2. Minimum Sample Size

Sample size calculation was considered to achieve the main objective of the study. The minimum sample size required to detect an effect size of 0.5 between the low and normal skeletal muscle mass older adults, a power of 80%, and a significance level of 0.05 is 75 subjects per group. Thus, the minimum sample size needed is 150 subjects. The calculation was done using G*Power version 3.0.10. The final sample consisted of 162 participants.

### 2.3. Nutritional and Dietary Assessment

Participants were asked to take an FFQ in Arabic for dietary assessment [21]. Data on frequencies of foods consumed and estimated quantities were converted into grams per day. Energy and nutrient intakes were estimated using Nutrilog^®^ 2.3 software. Lebanese food recipes were taken from the American University of Beirut database [21]. International food items and their nutrient values were taken from the United States Department of Agriculture (USDA) database and the ‘CIQUAL’ French food composition table [22,23]. Plant proteins included legumes such as beans, lentils, chickpeas, and green peas, as well as nuts, cereals and seeds. Animal proteins included red meat, poultry, fish, eggs and dairy. Personal interviews were performed with all subjects to collect their demographics and lifestyle-related data, previous medical history, and medication use. Participants were also asked if and how much they used dietary supplements and, if so, type of supplement, i.e., vitamin D, calcium, omega−3 polyunsaturated fatty acids, multivitamins, etc.

### 2.4. Strength Assessment

Handgrip strength (HGS) and the Short Physical Performance Battery (SPPB) were used to assess muscle strength and physical performance for all the participants. Handgrip strength was measured in the dominant hand with a Martin vigorimeter (Elmed, Addison), and the force was stated in kilograms [24]. The thresholds set for low muscle strength were <27 kg for men and <16 kg for women. Physical performance was assessed using the SPPB, which consists of three tests: (1) a standing balance test, including side-by-side, semi-tandem, and tandem stands; (2) a 6 m usual-pace walking test; and (3) the five-times sit-to-stand test. Low physical performance was defined as a participant unable to maintain a standing balance for 10 s, or a 6 m gait speed <0.8 m/s, or a 5-times sit-to-stand-test time ≥20 s. Each component was scored from 0 to 4 points according to the standardized SPPB scoring system. The three component scores were then summed to obtain a total SPPB score ranging from 0 to 12 points, with higher scores indicating better physical performance. The assessment was conducted by trained personnel following standardized procedures.

### 2.5. Statistical Analysis

Statistical analysis was performed using SPSS software version 27. The Chi–2 test was used to compare pairwise categorical variables, and effect size was estimated using Cramer’s V. The t-test for independent samples was used to compare two means, and effect size was estimated using Cohen’s *d*.

Multivariable logistic regression was performed to identify factors associated with low skeletal muscle mass. The dependent variable was low skeletal muscle mass, defined according to the sex-specific SMMI thresholds described above. Skeletal muscle mass or SMMI was not included as an independent predictor in the regression model, because these variables were used to define the outcome and their inclusion would result in circularity. The variables entered in the model were those that showed a *p* < 0.25 in the bivariate analysis [25]. Potential confounding factors considered in the analysis included demographic characteristics, lifestyle factors, and relevant comorbidities. The final model included the variables retained according to the predefined analytical strategy. The threshold for statistical significance was set at *p* < 0.05.

## 3. Results

Participants with lower skeletal muscle mass had significantly lower hand grip strength as well as SPPB scores. They also showed lower intakes of both animal and plant protein than those with normal skeletal muscle mass (Table 1).

The correlation matrix assessing factors associated with total, animal-source and plant-source protein intake showed that higher handgrip strength was significantly associated with higher animal protein intake, whereas higher SPPB scores were significantly but weakly associated with the total, animal-source and plant-source protein intake (Table 2).

### Multivariate Analysis

The results of the logistic regression showed that a higher handgrip strength (aOR = 0.92), a higher SPPB (aOR = 0.49), and a higher intake of animal protein (aOR = 0.87) or plant protein (aOR = 0.88) were significantly associated with lower odds of low skeletal muscle mass (Table 3).

To account for potential confounding factors, all multivariate analyses were adjusted for gender, smoking status, physical activity level, diabetes, cardiovascular disease (CVD), and hypertension. After adjustment for these covariates, the observed associations remained statistically significant, indicating that the relationship between exposure (handgrip strength, SPPB, animal protein and plant protein) and outcome (skeletal muscle mass) was independent of differences in these demographic, lifestyle and clinical characteristics.

## 4. Discussion

This study investigated the association between dietary protein sources and low skeletal muscle mass among Lebanese older adults. As expected, participants classified as having lower skeletal muscle mass had lower handgrip strength and physical performance compared with participants classified as having normal skeletal muscle mass. These differences are consistent with the clinical characteristics used to identify and characterize sarcopenia, and should therefore be interpreted primarily as expected phenotypic differences rather than independent findings of clinical relevance. Accordingly, here we place greater emphasis on the observed associations between animal-source and plant-source protein intake, and low skeletal muscle mass-related outcomes.

An important finding of the present study was that higher intakes of both animal- and plant-source protein were associated with lower odds of low skeletal muscle mass after multivariable adjustment. The association was somewhat stronger for animal-source protein than for plant-source protein. These findings are consistent with previous research suggesting that protein source may be associated with differences in muscle-related outcomes, although they should not be interpreted as evidence that animal protein causes greater preservation of muscle mass.

McCann et al. [26] recently performed a systematic review and meta-analysis of randomized controlled trials and reported that animal protein resulted in a greater increase in muscle mass than plant protein. The advantage of animal protein was evidenced in comparison to non-soy plant proteins and plant-based dietary interventions [26]. However, the authors found no significant differences between protein sources for muscle strength or physical performance outcomes. These findings suggest that protein source may be important for maintaining or increasing muscle mass and thus improving muscle function [26]. Furthermore, the trend observed in this study, i.e., that animal proteins may be associated with benefits for muscle mass, is consistent with recent meta-analyses showing that consumption of animal protein leads to greater gains in lean body mass compared to plant protein [27,28]. This advantage of animal-source protein has largely been attributed to its higher digestibility and more favorable essential amino acid profile, in particular the content of leucine which plays a central role in stimulating muscle protein synthesis [26,28,29]. Animal-source proteins such as whey, milk, eggs, meat, chicken and fish normally provide a more complete amino acid profile than most plant proteins [26]. Evidence from observational studies and randomized controlled trials further supports this hypothesis: Houston et al. [30] showed that higher total protein intake was associated with reduced lean mass loss over a three-year period in older adults, and Daly et al. [17] found that combining resistance training with higher consumption of lean red meat significantly increased muscle mass and strength in elderly women.

This study has several implications for clinical practice. Older adults at risk of sarcopenia may benefit from prioritizing protein sources rich in essential amino acid profile. While animal proteins appear to provide a greater anabolic stimulus [31,32], plant-based proteins have also been associated with comparable anabolic responses to some animal proteins, particularly when consumed in sufficient quantities and combined with resistance exercise [28,33]. Plant-based proteins offer several nutritional and health advantages but may be less effective on a gram-for-gram basis in terms of stimulating muscle protein synthesis [34]. Many plant proteins contain lower concentrations of one or more essential amino acids. For instance, cereals are often limited in lysine and legumes are limited in methionine [34], and these limitations may lead to a lower anabolic response compared with animal-source protein. However, the strategic combination of complementary plant proteins, such as legumes and grains, can improve amino acid balance and help meet essential amino acid requirements. Further research is needed to investigate the associations between the consumption of emerging plant protein sources and muscle health, as well as the potential benefits of combining complementary plant proteins to achieve a more balanced amino acid profile and improve the nutritional adequacy of protein intake. Moreover, long-term clinical outcomes should be considered in sarcopenic older adults.

The results of this study have important clinical implications in terms of factors associated with the presence and management of low skeletal muscle mass in older adults. Healthcare professionals, including physicians, dietitians, nurses and physiotherapists, should address the source of dietary protein in addition to total protein intake when assessing older adults at risk of muscle loss. Evaluating the sources and allocation of protein between meals may help classify individuals with inadequate protein intake or an unfavorable amino acid profile and thus direct them towards specific nutritional interventions. Dietitians can encourage the inclusion of protein sources rich in essential amino acids and promote complementary combinations of plant proteins. Furthermore, combining an adequate protein intake with resistance exercise could grant greater benefits for preserving muscle mass, muscle strength and physical performance. These strategies may facilitate earlier nutritional intervention and provide a more comprehensive approach to sarcopenia prevention and management in older adults. However, although we identified associations between animal and plant protein intake and sarcopenia-related outcomes, our findings do not establish causality.

There are several limitations to the findings of this work. First, the cross-sectional design prevents determination of temporal or causal relationships. It is therefore not possible to conclude that higher animal- or plant-source protein intake leads to greater skeletal muscle mass. Reverse causation is also possible; for example, individuals with better muscle health may have different dietary patterns or greater capacity to prepare and consume protein-rich foods. Second, dietary intake was assessed using self-reported dietary data, which may be subject to recall and reporting bias. Third, although we considered potential confounding factors such as age, gender, total protein intake, physical activity and comorbidities, we cannot completely rule out residual confounding factors. Fourth, the recruitment of participants from selected Lebanese communities may limit the generalizability of the findings to the broader Lebanese older-adult population.

Another important methodological consideration is the definition of the primary outcome. The present study classified participants according to SMMI and therefore assessed low skeletal muscle mass, rather than applying a complete contemporary diagnostic algorithm for sarcopenia. HGS and SPPB were available and were assessed as complementary measures of muscle strength and physical performance, but they were not incorporated into the primary classification. Future studies should consider applying comprehensive diagnostic criteria incorporating muscle strength, muscle quantity or quality, and physical performance.

## 5. Conclusions

In conclusion, although both animal- and plant-source proteins contribute to muscle health in older people, current evidence suggests a slightly stronger positive association between animal-source protein and muscle mass, muscle strength and muscle function, which are the key components of sarcopenia prevention. This advantage is most likely attributable to the superior amino acid profile and digestibility of animal protein. Nevertheless, plant-based protein sources can provide similar benefits when consumed in appropriate amounts. Therefore, both the amount and type of protein consumed should be considered, rather than the protein source alone.

However, the observational and cross-sectional nature of the study prevents causal inference, and the findings should not be interpreted as evidence that animal protein prevents sarcopenia or causes greater preservation of muscle mass. Longitudinal and interventional studies are now needed to determine whether differences in animal- and plant-source protein intake contribute to changes in skeletal muscle mass, muscle strength or physical performance over time, and to clarify the potential role of overall dietary patterns in the maintenance of muscle health among older adults.

## Figures and Tables

**Table 1:** Bivariate analysis of factors associated with normal versus low skeletal muscle mass.

Variable	Normal Skeletal Muscle Mass	Low Skeletal Muscle Mass	Total	*p*	Effect Size
Gender				0.536	0.049
Male	36 (48.0%)	39 (52.0%)	75 (100%)		
Female	46 (52.9%)	41 (47.1%)	87 (100%)		
Smoking				0.698	0.040
No	64 (49.6%)	65 (50.4%)	129 (100%)		
Yes	18 (54.5%)	15 (45.5%)	33 (100%)		
Physical Activity				0.013	0.039
No	25 (30.2%)	64 (81.5%)	89 (100%)		
Yes	57 (69.8%)	16 (18.5%)	73 (100%)		
Diabetes				0.803	0.020
No	58 (50.0%)	58 (50.0%)	116 (100%)		
Yes	24 (52.2%)	22 (47.8%)	46 (100%)		
Hypertension				1	0.001
No	39 (50.6%)	38 (49.4%)	77 (100%)		
Yes	43 (50.6%)	42 (49.4%)	85 (100%)		
Coronary Artery Disease				1	0.009
No	65 (50.4%)	64 (49.6%)	129 (100%)		
Yes	17 (51.5%)	16 (48.5%)	33 (100%)		
Age (Years)	71.66 ± 5.44	71.66 ± 5.13	71.66 ± 5.27	0.996	0.001
Body Mass Index (kg/m^2^)	26.90 ± 4.25	26.10 ± 4.53	26.51 ± 4.39	0.246	0.183
Fat Mass Percentage	32.22 ± 4.34	32.97 ± 3.67	32.59 ± 4.03	0.236	0.187
Hand Grip Strength (kg)	23.76 ± 4.72	21.25 ± 5.61	22.52 ± 5.31	**0.003**	0.484
Short Physical Performance Battery	8.61 ± 1.06	7.26 ± 1.57	7.94 ± 1.50	**<0.001**	1.006
Carbohydrates (Grams/Day)	207.02 ± 43.57	196.93 ± 28.37	202.04 ± 37.09	0.082	0.274
Animal Proteins (Grams/Day)	33.33 ± 6.10	29.18 ± 4.74	31.28 ± 5.84	**<0.001**	0.760
Plant Proteins (Grams/Day)	31.17 ± 4.72	27.70 ± 4.38	29.45 ± 4.86	**<0.001**	0.762
Animal Protein Intake (g/kg/Day)	0.45 ± 0.11	0.40 ± 0.09	0.43 ± 0.10	**<0.001**	0.561
Plant Protein Intake (g/kg/Day)	0.43 ± 0.09	0.38 ± 0.09	0.40 ± 0.10	**0.002**	0.493
Fat (Grams/Day)	36.22 ± 10.53	38.15 ± 9.38	37.17 ± 10.00	0.220	0.193

Numbers in bold indicate significant *p* values. Effect size was determined by Cramer’s V or Cohen’s *d*.

**Table 2:** Correlation matrix of continuous scores.

	1	2	3	4	5
1. Total Protein Intake	1				
2. Animal Protein	0.80 ***	1			
3. Plant Protein	0.69 ***	0.11	1		
4. Handgrip Strength	0.13	0.19 *	0.02	1	
5. Short Physical Performance Battery	0.28 ***	0.19 *	0.23 **	0.16 *	1

* *p* < 0.05; ** *p* < 0.01; *** *p* < 0.001.

**Table 3:** Multivariate analysis: logistic regression of factors associated with low skeletal muscle mass (Nagelkerke *R*^2^ = 0.493).

*Va* *riable*	*p*	*aOR*	*95% CI*
Fat Mass Percentage	0.676	1.02	0.92–1.13
Handgrip Strength (kg)	**0.031**	0.92	0.84–0.99
Short Physical Performance Battery	**<0.001**	0.49	0.35–0.69
Carbohydrates (Grams/Day)	0.465	1.00	0.99–1.01
Animal Protein (Grams/Day)	**<0.001**	0.87	0.81–0.94
Plant Protein (Grams/Day)	**0.005**	0.88	0.80–0.96
Fat (Grams/Day)	0.086	1.04	1.00–1.08

Numbers in bold indicate significant *p* values. aOR = adjusted odds ratio; CI = confidence interval.

## Data Availability

The dataset generated and analyzed during this study is available in Supplementary Materials (Supplementary File S1).

## Supplementary Materials

The following supporting information can be downloaded at: https://www.mdpi.com/article/10.3390/nu18203333/s1, Supplementary File S1: Database of the participants.
